# Nano-hydroxyapatite in oral care cosmetics: characterization and cytotoxicity assessment

**DOI:** 10.1038/s41598-019-47491-z

**Published:** 2019-07-30

**Authors:** Catarina C. Coelho, Liliana Grenho, Pedro S. Gomes, Paulo A. Quadros, Maria H. Fernandes

**Affiliations:** 1 Instituto de Investigação e Inovação em Saúde, U.Porto, Porto Portugal; 2 Instituto de Engenharia Biomédica, U.Porto, Porto Portugal; 3 Faculdade de Engenharia, U.Porto, Porto Portugal; 4 FLUIDINOVA, S.A., Maia, Portugal; 5 Laboratory for Bone Metabolism and Regeneration, Faculdade de Medicina Dentária, U.Porto, Porto Portugal; 6 LAQV/REQUIMTE, U.Porto, Porto 4160-007 Portugal

**Keywords:** Nanotoxicology, Nanoparticles

## Abstract

Nano-hydroxyapatite has been used as an oral care ingredient, being incorporated in several products for the treatment of dental hypersensitivity and enamel remineralisation. Despite its promising results, regulatory and safety concerns have been discussed and questioned by the European Scientific Committee on Consumer Safety (SCCS) regarding the usage of hydroxyapatite nanoparticles in oral care products. In this work, a commercially available nano-hydroxyapatite was characterized and its cytocompatibility towards human gingival fibroblasts was evaluated, as well as its irritation potential using the *in vitro* HET-CAM assay. All the conditions chosen in this study tried to simulate the tooth brushing procedure and the hydroxyapatite nanoparticles levels normally incorporated in oral care products. The commercial hydroxyapatite nanoparticles used in this study exhibited a rod-like morphology and the expected chemical and phase composition. The set of *in vitro* cytotoxicity parameters accessed showed that these nanoparticles are highly cytocompatible towards human gingival fibroblasts. Additionally, these nanoparticles did not possess any irritation potential on HET-CAM assay. This study clarifies the issues raised by SCCS and it concludes that this specific nano-hydroxyapatite is cytocompatible, as these nanoparticles did not alter the normal behaviour of the cells. Therefore, they are safe to be used in oral care products.

## Introduction

Hydroxyapatite is the main mineral component of vertebrate bone and tooth, where this calcium phosphate is present as nanoscospic crystals. Synthetic hydroxyapatite has been extensively used for decades in biomedical applications due to its excellent biocompatibility and osteogenic capacity^1^. More recently, hydroxyapatite nanoparticles (HA-NP) have been incorporated in oral care products to treat dentin hypersensitivity (DH) and promote enamel remineralisation^2–7^. DH is a frequent oral health condition that affects up to 30% of the adults in a certain period of their lives^8^. In patients with periodontitis, the prevalence of DH can even reach 98%^9^. In this condition, dentine tubules are exposed to the oral environment due to the presence of lesions in the tooth enamel. As dentin tubules are open to the external environment, they are submitted to different stimuli that can reach the pulp nerves, resulting in a short and sharp pain^10^. HA-NP are able to reduce the pain associated with DH since nanoparticles are small enough to enter and fill the dentin tubules^11^. The nanoparticles precipitate inside the tubules and consequently block the interaction of the nerves with the external stimuli^12^. HA-NP can also precipitate onto the enamel surface and remineralise initial enamel caries, resulting in the formation of a new apatite layer with increased enamel surface hardness and therefore preventing tooth decay^13^. These positive results can be related to HA-NP high similarity with tooth enamel, in terms of morphology and crystal structure^14^.

As HA-NP possess such good performance in the treatment of DH, a rising number of toothpastes and mouthwashes containing this nanomaterial emerged in the market in the last decade. However, the incorporation of these nanoparticles has raised several issues related with their potential health risk for the consumers, leading the European Scientific Committee on Consumer Safety (SCCS) to questioning the safety of these nanoparticles as an oral care ingredient in their adopted Opinion SCCS/1566/15^15^. This document contains a safety assessment with a comprehensive analysis about the toxicological potential of these nanoparticles as a cosmetic ingredient. Even with such exhaustive investigation, SCCS was not able to draw a final conclusion about the safety of HA-NP. One of the reasons was the insufficient characterization of the nanoparticles, as the toxicological effect may be related with features such as size and morphology. Therefore, SCCS requires that the toxicological evaluations must be combined with a characterization of each ingredient individually^15^.

In this work, commercially available HA-NP were evaluated for cytotoxicity, envisaging their application in the treatment of dentin hypersensitivity. HA-NP particles were characterized in terms of morphology, size, surface area and chemistry. *In vitro* cytotoxicity to human gingival fibroblasts (HGF) was evaluated for cell viability, metabolic activity, cell morphology, F-actin cytoskeleton organization, rate of apoptosis and generation of reactive oxygen species (ROS). Additionally, the irritant potential of these particles was assessed by the *in vitro* hen’s egg test on the chorioallantoic membrane (HET-CAM) assay. All assays were performed in conditions attempting to mimic tooth brushing procedure in terms of relevant cell type, exposure time and HA-NP levels incorporated in oral care products, in order to answer the concerns raised by the SCCS regarding the use of this nanomaterial in oral care products.

## Results

### HA-NP characterization

**Figure 1:**
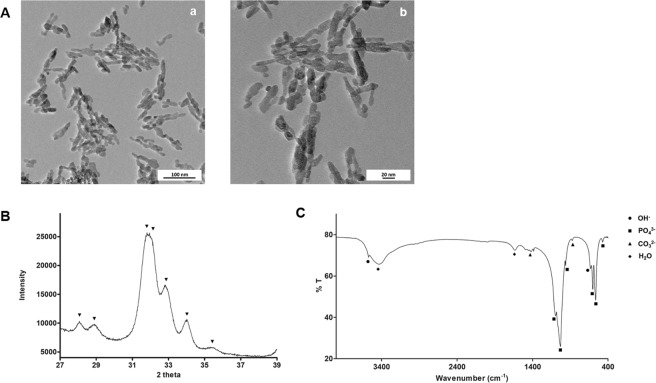
Characterization of HA-NP. (**A**) TEM micrographs. Scale bar: 100 nm (a) and 20 nm (b); (**B**) XRD spectrum with the patterns for hydroxyapatite and (**C**) FTIR spectrum labelled with hydroxyapatite characteristic peaks.

The morphology and size of the nanoparticles were analysed using TEM, Fig. 1A. Particles have a rod-like shape, with an average length of 26 ± 11 nm and an average width of 9 ± 2 nm, which confirms the nanometric size of the hydroxyapatite particles. BET surface area analysis showed a value of 128.4 ± 5.5 m^2^/g.

Chemical characterization was performed by XRD and FTIR. The XRD spectrum did not show any unexpected phases for hydroxyapatite, as peaks position are in agreement with ISO 13779-3 requirements, Fig. 1B. According to this international standard, hydroxyapatite is identifiable by peaks 202, 210 and 211, which correspond to 34, 28 and 31 at 2θ. FTIR was also performed to evaluate the presence of functional groups at the molecular level, Fig. 1C. The spectrum reveals the hydroxyapatite characteristic peaks, namely phosphate groups at 473, 564, 603, 962, 1032 and 1094 cm^−1^. In addition, the bands present at 632 and 3572 cm^−1^ are related with OH^−^ vibrational and stretching modes. Moreover, the existence of peaks at 876, 1421 and 1454 is due to the presence of carbonate group^16^. The latter can result from the incorporation of atmospheric CO_2_ during hydroxyapatite processing^17^. Finally, the peak at 1633 cm^−1^ and the broad band at 3200–3600 cm^−1^ indicate the presence of adsorbed water on the material.

### Cytotoxicity of HA-NP

#### Cell viability

**Figure 2:**
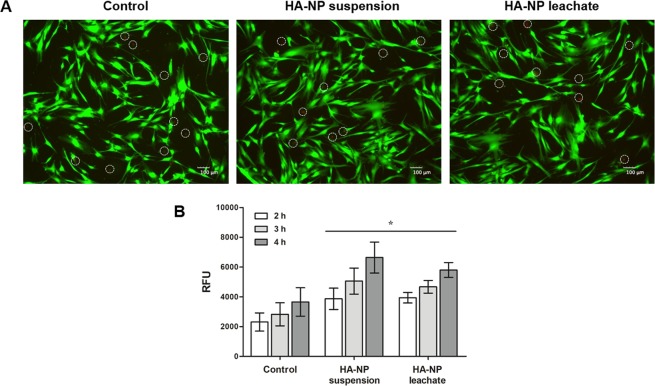
Cell viability and metabolic activity. (**A**) Live/dead cell staining after exposure to HA-NP suspension or leachate for 1 h. Live cells stain green and dead cells stain red (marked with a circle for better localization). Scale bar: 100 µm; (**B**) Metabolic activity of cells exposed to HA-NP suspension or leachate for 2, 3 and 4 h. *Significantly different from control (non-treated cells) for each time-point, (p < 0.05).

Cell viability was evaluated by live/dead staining assay after 10 min and 1 h of exposure to the HA-NP suspension or leachate. Figure 2A shows that cell viability was not affected with both samples. Fibroblasts were well adhered and spread, with their typical elongated morphology (green coloured). Only few dead cells were visible (red coloured) in the control and in the exposed cells.

#### Metabolic activity

Cell metabolic activity, evaluated by the resazurin assay, and tested after 2, 3 and 4 h of exposure to HA-NP suspension and leachate is represented in Fig. 2B. For all the time points tested, HA-NP increased the metabolic activity compared to that observed in non-treated cells.

#### F-Actin cytoskeleton organization and Mitochondria tracking

**Figure 3:**
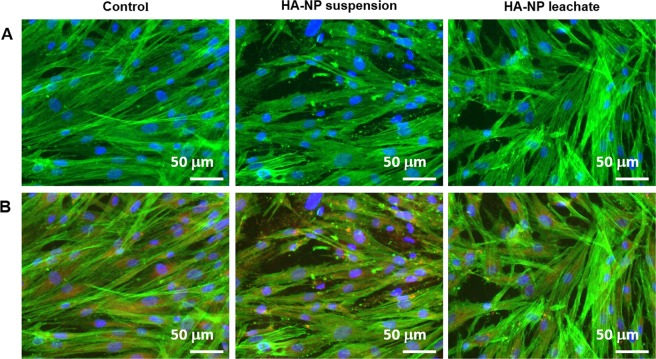
Staining of F-actin, mitochondria and nucleus. (**A**) F-actin cytoskeleton organization after exposure to HA-NP suspension or and HA-NP leachate for 4 h; (**B**) F-actin cytoskeleton, mitochondria and nucleus staining after exposure to HA-NP suspension or and HA-NP leachate for 4 h. F-actin (green), mitochondria (red) and nucleus (blue). Scale bar: 50 µm.

On the cytoskeleton fluorescence images, cells exposed to the HA-NP suspension or leachate displayed a healthy appearance, similar to the control, i.e. elongated morphology, prominent nucleus, well-organized F-actin filamentous stress fibres and intense staining in the cell limits. Figure 3A shows representative images for cells exposed for 4 h. Similar appearance was exhibited for cells exposed for 1 h (not shown). Mitochondria localization was evaluated using a specific tracking dye, after 1 and 4 h exposure. Fluorescence images were similar in control cultures and those exposed to the HA-NP suspension and leachate. Mitochondria showed mostly perinuclear localization. Images of cells stained for F-actin cytoskeleton, mitochondria and nucleus clearly suggest the presence of healthy cells, Fig. 3B (4 h exposure).

#### Apoptotic index

**Figure 4:**
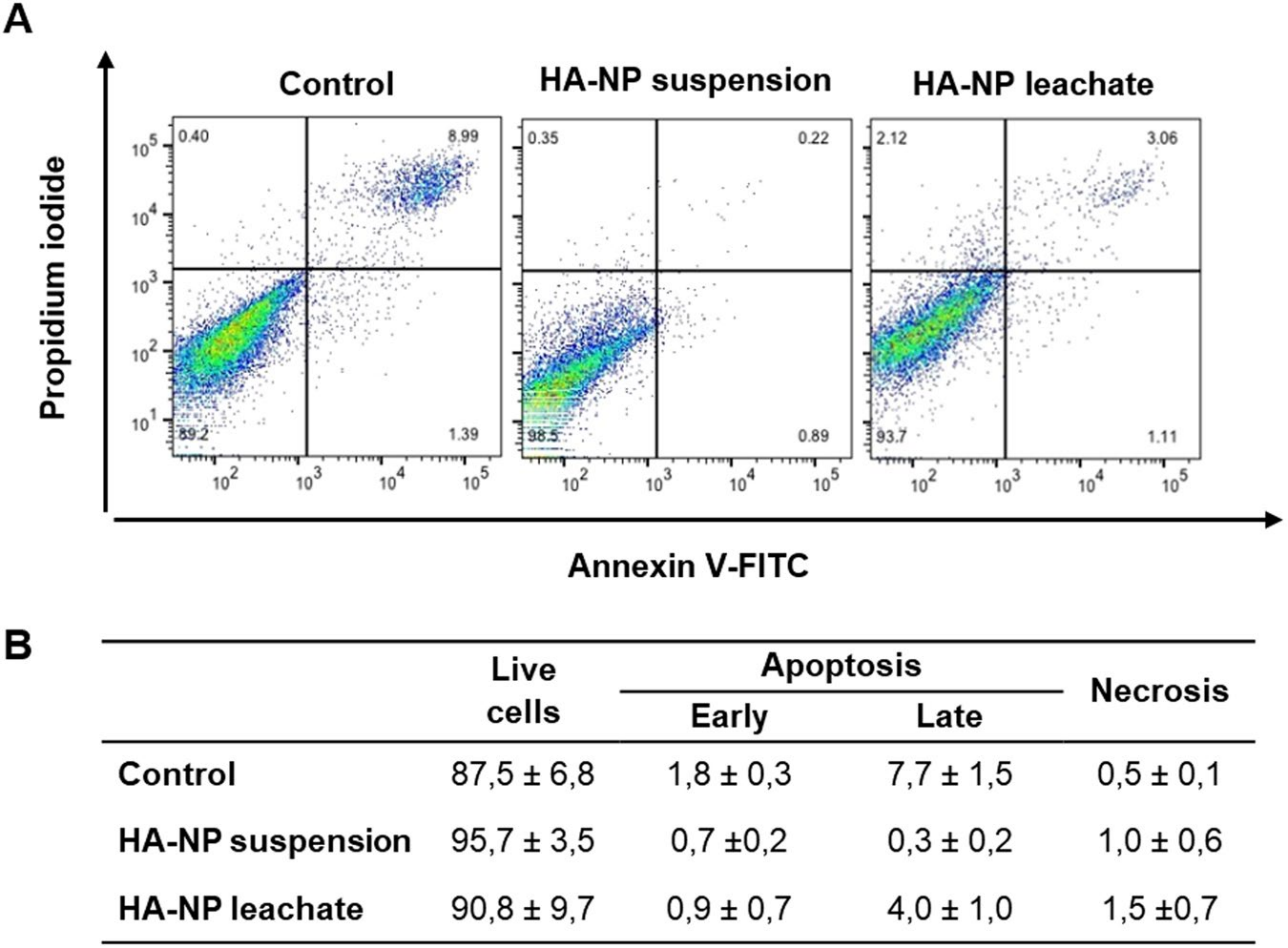
Flow cytometry results with representative histograms (**A**) and quantification (**B**) of the effect of HA-NP suspension or leachate on apoptosis in HGF, after 4 h of exposure.

The percentage of early and late apoptotic cells, analysed by flow cytometry, was very low and similar in control cells and those exposed to the HA-NP suspension and leachate. Figure 4 shows representative histograms and the quantification values in cultures exposed for 4 h.

#### Production of ROS

**Figure 5:**
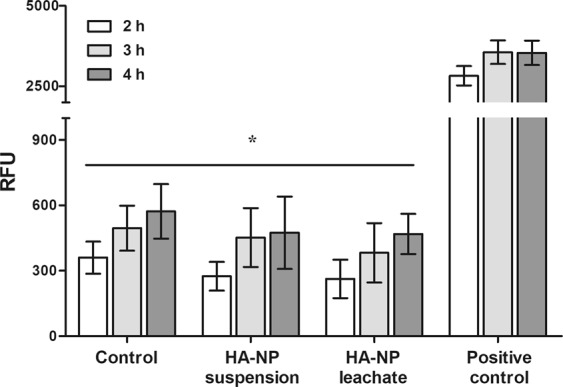
ROS production after HGF exposure to HA-NP suspension or leachate for 4 h. Cells were also treated with TBPH as positive control and non-treated cells were also used as control. *Significantly different from positive control for each time-point, (p < 0.05).

Both the HA-NP suspension and the leachate did not induce the production of ROS in the fibroblasts, after being exposed for 2, 3 and 4 h. As demonstrated in Fig. 5, the fluorescence of cells treated with HA-NP was always significantly lower than the positive control (cells treated with TBHP) and similar to that on non-treated cells.

#### The irritation potential by the HET-CAM Assay

**Figure 6:**
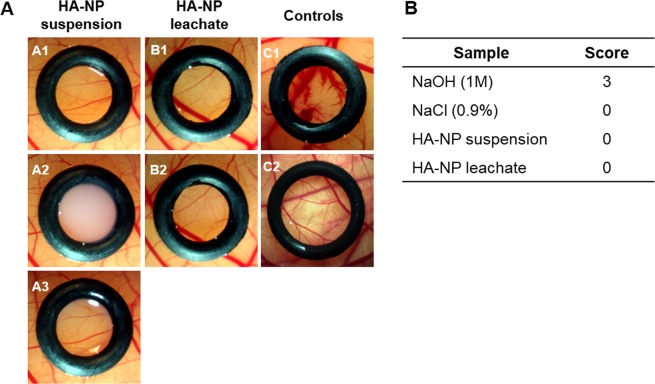
*In vitro* HET-CAM assay. (**A**) Macroscopic features of CAM vasculature after a 5 min exposure time to HA-NP suspension or leachate. HA-NP suspension: before the exposure (A1), with the suspension (A2) and after removing the sample (A3); HA-NP leachate: before (B1) and after (B2) the exposure; positive control (NaOH, 0.5 M) (C1) and negative control (NaCl, 0.9%) (C2); (**B**) Classification table with irritation score for the *in vitro* HET-CAM assay.

On the irritation potential assay, the HA-NP suspension and leachate did not induce noticeable adverse vascular alterations on the CAM. The appearance was similar to that observed in the negative control (treatment with NaCl, 0.9%) and greatly different of that on the positive control (treatment with NaOH, 0.5 M). Representative images and semi-quantitative score values are shown in Fig. 6.

## Discussion

The main goal of this study was to characterize and evaluate the cytocompatibility of HA-NP, more specifically a particular commercial HA-NP product, used as an ingredient in oral care.

There is not a consensus regarding the influence of the physico-chemical properties on the nanoparticle toxicity. Particularly for calcium phosphates, Epple referred that the calcium phosphate phase or the particle shape may not have a significant influence on the biological response^18^. On the other hand, it is also know that some characteristics of HA particles may influence cytotoxicity^19^. In the present study, HA-NP particles were characterized in terms of morphology, size, chemical composition and surface area as these aspects may be relevant for nanoparticle toxicity^15^.

Although this is well established, published studies on the performance of oral products containing hydroxyapatite nanoparticles did not address this issue^15^. The tested particles exhibited rod-shape appearance, a morphology that is reported to be associated to a higher cytocompatibility, in comparison with other shapes such as needle-like^20,21^. In fact, the SCCS opinion on nano-hydroxyapatite reports different studies that mention higher toxicity for needle-shaped nanoparticles^15^. Effects such as genotoxicity, changes in liver function, induction of oxidative stress and possible systemic availability led SCCS to suggest avoiding the use of needle-shape hydroxyapatite nanoparticles in cosmetics. In terms of surface area, the HA-NP used in this study present a high surface area (128.4 ± 5.5 m^2^/g) which increases the potential for the interactions with the cellular structures and the surrounding extracellular fluids. As expected, XRD and FTIR analyses confirm the chemical purity of these particles.

To perform the *in vitro* cytotoxicity assays, it was taken into consideration several aspects of the tooth brushing procedure such as exposure time, levels of HA-NP incorporated in toothpastes and relevant cell type. Regarding the duration, the average time for tooth brushing is 2 minutes^22,23^. However, longer exposure times were tested in this work, as a precaution and to have a more clear idea of the effect of the particles that may remain inside the oral cavity after brushing. As mentioned before, the maximum incorporation of nanoXIM^®^ hydroxyapatite ingredient in toothpastes is 20%. Therefore, the suspension prepared had the highest possible concentration of these nanoparticles in commercial toothpastes, so the tests were performed in the worst case scenario. Finally, it was decided to prepare two types of 20% nanoXIM^®^ samples, a suspension and a leachate. The nanoXIM^®^ suspension mimics the dispersion of the nanoparticles inside the oral cavity during tooth brushing. On the other hand, the hydroxyapatite nanoparticles may release some ions during brushing and the effect of those ions was studied with the nanoXIM^®^ leachate samples. Cytotoxicity assays were conducted with human gingival fibroblasts, a relevant cell type regarding the exposure to the toothpaste brushing.

Tooth brushing only takes a few minutes and it is valuable to access the cell viability for short time points. Considering this, firstly a live/dead fluorescent staining was performed after 10 minutes exposure to HA-NP suspension and leachate. Cell viability was not affected after this exposure time. Also, an assay conducted with a longer time (1 h) gave similar information.

The live/dead assay provides only gross information regarding the cytotoxicity potential as the underlying mechanism is set up on the integrity of the cellular membrane, thus detecting only the presence of live and dead cells. In this way, cell behaviour was also assessed by the resazurin assay that is useful to assess mitochondrial metabolic activity, as the irreversible reaction of resazurin to resorufin is proportional to aerobic respiration. Increased values were observed in the fibroblasts exposed to HA-NP suspension and leachate. Some hypothesis of explanation might be anticipated. It is known that HA-NP are readily internalized by an endocytic mechanism being localized in intracellular vesicles^18^. Dissolution of HA-NP in the acid environment of the lysosomes might occur with the formation of calcium and phosphate ions, which diffuse into the cytoplasm^18^. An increase in the levels of intracellular calcium might be expected, taking into account the relatively high levels of HA-NP used (3.1%). Calcium ion is a key modulator of cellular activity including the mitochondrial function^24,25^, and the above mechanism might explain the observed increased metabolic activity^18^. However, a variety of studies strongly suggests that this effect might be temporary because cells are able to clear the calcium from the cytoplasm within a few hours^18^.

Mitochondria are the major site of energy production for cell survival being involved in many vital functions such as calcium signalling, cell growth and cell differentiation. They also have a key role in cell death including apoptotic and necrotic mechanisms^26^. In the present work, the HA-NP suspension and leachate appeared not to present mitochondrial deleterious effects, in an immunostaining assay that localizes mitochondria based on its membrane potential. This is a relevant finding considering the mitochondria role in the cell biology, and that it is now known that these organelles are highly tuned and prone to malfunction in response to even moderate stress^26^. In spite of the increased values of metabolic activity observed in the resazurin assay on the exposed fibroblasts, this appeared not to be associated with deleterious effects infringed to mitochondria.

Similar to the mitochondria, the F-actin cytoskeleton is a critical cell structure. It is crucial not only in cellular functions like division, gene expression, intracellular transport and signalling pathways, but also in motility and mechano-transduction mechanisms related with differentiation events^27,28^. Since it provides structural stability and it cooperates actively with cellular internal needs and response to surrounding environment, F-actin cytoskeleton is considered an early indicator of cytotoxicity^29^. Deleterious effects on this structure were not evident in the immunohistochemical cell staining. Images of triple staining (cytoskeleton, mitochondria and nucleus) are consistent with a healthy appearance of the exposed fibroblasts.

Deleterious effects to the cells may trigger the process of apoptosis, an energy-dependent and highly regulated process. Cell senses external and internal stress and, in response, activates the caspase cascade. In line with the previous assays, the percentage of apoptotic cells was very low in the fibroblasts exposed to the HA-NP suspension or leachate.

The evaluation of ROS levels is another relevant test to assess the cytotoxic potential of the nanoparticles^18^. ROS levels were measured using 2′,7′ – dichlorofluorescin diacetate (DCFDA, also known as H2DCFDA), one of the most widely used fluorescent probes to measure the redox state of a cell. For all the time point tests, both HA-NP suspension and leachate did not induce the production of ROS in the fibroblasts. This agrees with results in several cell types contacting with high levels of HA-NP showing some degree of apoptosis but no evidence of ROS production^20^.

The HET-CAM assay is a versatile and sensitive *in vitro* assay that uses fertilized hen eggs. The CAM membrane separates the embryo from the internal airspace and is non-innervated and highly vascularized, responding to injury in a similar way as mucosal and subcutaneous tissue^30^. In this work, both the HA-NP suspension and leachate did not induce detectable signs of irritancy.

Together, the observed results beginning with a basic live/dead assay, followed by assays able to detect more subtle cytotoxic effects that may compromise cell proliferation and function, and completed with an *in vitro* irritation assay, evidenced the absence of toxicity of the tested HA-NP particles in conditions focused in a topic use in the oral cavity. This methodology provided complementary information for more reliable cytotoxicity data on this specific ingredient, answering to some of the concerns raised by SCCS/1566/15 on the safety of HA-NP^15^. As referred above, the tested HA-NP concentration, both the particles and leachable products, is most probably higher than the ones that will contact with the oral tissues during tooth brushing, due to the immediate dilution with saliva, and continuous oral clearance. Also, swallowed HA particles are immediately dissolved in the acid environment of the stomach^18,31^, and do not pose any safety concern.

In summary, this study evaluated the cytotoxic potential of a commercially available HA-NP suspension, in conditions attempting to mimic tooth brushing procedure in terms of relevant cell type, exposure time and maximum HA-NP levels incorporated in oral care products. A 3.1% HA-NP suspension (to mimic the contact with the particles) and its leachate (to mimic the contact with the particles’ leachable products) were tested using complementary cytotoxicity assays addressing cell structures sensitive to potential stress induced stimulus. *In vitro* results on cell viability, F-actin cytoskeleton organization, mitochondria tracking, apoptotic index and ROS formation converged to evidence a high cytocompatibility of the HA-NP towards human gingival fibroblasts. The *in vitro* HET-CAM assay proved that HA-NP were devoid of any irritation potential. Considering that the cytotoxicity assays were performed in the worst predictable exposure scenario, the use of these HA-NP in oral care products does not pose any safety concern.

## Materials and Methods

### Hydroxyapatite nanoparticles

A commercially available formulation of HA-NP (15.5% suspension of hydroxyapatite nanoparticles in water: nanoXIM^®^ hydroxyapatite suspension, FLUIDINOVA, S.A., Portugal) was used in this study and it was sterilized by autoclaving (120 °C, 15 minutes).The sterilization process did not alter the physico-chemical properties of the material (Supplementary Figs 1–3).

### Nanoparticle characterization

#### Transmission electron microscopy

Transmission electron microscopy (TEM) was used to characterize the HA-NP in terms of morphology and size. A suspension of nanoparticles in ethanol was immersed in an ultrasonic bath for 30 minutes for particle dispersion. Then, samples were mounted on copper grids and left to dry inside a desiccator. Visualization was carried out on a Hitachi HD2700 equipment and the images were analysed using ImageJ Fiji software. For particle size measurement, 100 nanoparticles (n = 100) from 3 different images acquired with TEM. Each particle was measured in its longer and shorter dimension.

#### Brunauer–Emmett–Teller surface area

The specific surface area of the nanoparticles was analysed using Brunauer-Emmett-Teller (BET) method with a Quantachrome NOVA 2000e equipment. For that purpose, the hydroxyapatite nanoparticles suspension was dried overnight at 105 °C to remove the water and milled to obtain a fine powder. Sample degasification was performed at 300 °C during 2 hours.

#### X-ray diffraction

For chemical characterization analysis, the HA-NP suspension was dried overnight at 105 °C to remove the water and milled to obtain a fine powder. The hydroxyapatite phase composition was evaluated by x-ray diffraction (XRD) using a PANanalytical Empyrean diffractometer (Step scan time: 198.6 s; step size: 0.0130 °2θ and 2θ range: 27–39°). The hydroxyapatite phase purity was appraised taking into consideration the international standard ISO 13779-3: Implants for surgery – Hydroxyapatite – Part 3: Chemical analysis and characterization of crystallinity and phase purity.

#### Fourier transform infrared spectroscopy

To perform Fourier transform infrared spectroscopy (FTIR), the hydroxyapatite suspension was dried and milled. Then, the resulting powder was analysed as a KBr pellet using a Perkin Elmer Frontier spectrometer, with a resolution of 4 cm^−1^, frequency region from 400 to 4000 cm^−1^ and 100 scans accumulated per sample.

### *In vitro* cytotoxicity assays

#### Sample preparation

To perform the *in vitro* tests, two types of samples were prepared: a HA-NP suspension and its leachate. According to the HA-NP manufacturer, the maximum incorporation of this ingredient (15.5% HA-NP suspension) in oral care products is 20%^31^. For that reason, it was decided to use this single maximum dose as it represents the worst case scenario. Considering this, a 20% suspension was made by diluting 0.2 grams of well-homogenized suspension per millilitre of Alpha Minimum Essential Medium (α-MEM) without phenol red (Gibco) resulting in a 3.1% HA-NP suspension. Afterwards, the suspension was vortexed at 25 Hz during 5 seconds for homogenization. In addition, the 3.1% HA-NP suspension was centrifuged at 4000 g during 10 minutes and the supernatant was spared to evaluate the effect of possible leachable products (HA-NP leachate). Both HA-NP samples (suspension and leachate) were freshly prepared just before the *in vitro* tests and the same concentration was used for all tests. The times and methods were chosen in an attempt to mimic the tooth brushing procedure.

#### Cell culture

Human gingival fibroblast (HGF) cells were maintained in α-MEM (Sigma-Aldrich) supplemented with 10% (v/v) fetal bovine serum (FBS, Gibco), 2.5 µg/mL amphotericin B (Gibco), 100 IU/mL penicillin and 100 µg/mL streptomycin (Gibco). At about 70–80% confluence, cells were enzymatically detached using a trypsin - EDTA solution. For all the experiments, cells were seeded at a density of 3 × 10^4^ cells/cm^2^. After 24 hours incubation, cells were exposed to the particles suspension or the leachate for periods up to 4 hours. Cells cultured in the absence of the HA-NP samples were used as control. Cultures were incubated at 37 °C in an atmosphere of 5% CO_2_. Cell response was evaluated as follows.

#### Live/dead cell staining

A live/dead cell staining was performed to evaluate cell viability after a short exposure to the HA-NP. This assay simultaneously labels live and dead cells with fluorescent dyes (calcein and propidium iodide, respectively). Calcein AM is a non-fluorescent hydrophobic molecule that easily permeates the intact membrane of live cells and is converted by intracellular esterases to a hydrophilic and strongly fluorescent molecule (calcein, green) that is retained in the cell cytoplasm. Propidium iodide stain dead cells red; it is membrane-impermeable but enter cells with compromised plasma membranes and binds to DNA with high affinity, a process that is associated with a significant increase in the fluorescence intensity.

To perform this assay, 24 hours post-seeding, cells were washed with phosphate buffered saline (PBS) and then incubated with HA-NP suspension or leachate for 10 min and 1 h. After, the HA-NP samples were removed and cells were incubated with Calcein AM (2 µl/mL, Sigma-Aldrich) and propidium iodide (PI, 50 µl/mL, BD Biosciences) solution during 30 min at 37 °C, protected from light. Non-treated cells were used as a control. Fluorescence was observed using an inverted fluorescence microscope (Axiovert 200 M, Zeiss) with green (488 nm) and red (594 nm) filters. Image processing was performed using AxioVision Software from Zeiss.

#### Metabolic activity

Metabolic activity was assessed using Alamar Blue® (resazurin) assay. Resazurin is a blue dye that is reduced in mitochondria to the pink coloured and highly red fluorescent resorufin.

Cells were cultured for 24 h in 96-well plates, the culture medium was removed and the cell layer was washed with PBS. Afterwards, 100 µL of HA-NP suspension or leachate were added to the cultures. Immediately after, 10% (v/v) of resazurin (0.1 mg/mL, Sigma-Aldrich) was added to the medium, and the metabolic activity was assessed after 2, 3 and 4 h of incubation. Non-treated cells were used as control. Fluorescence was measured at 530 nm excitation and 590 nm emission using a microplate reader (SynergyMix, BioTek) with Gen5 1.09 Data Analysis Software. Results are expressed as relative fluorescent units (RFU), for each time point.

#### Mitochondria tracking and F-Actin cytoskeleton

After 24 h of culture, cells were exposed to HA-NP suspension or leachate for 1 and 4 hours. The cell layer was then washed once with warm PBS and the mitochondria specific tracking dye, MitoSpy™ Red CMXRos (250 nM, BioLegend), was added and cultures were incubated for 30 min at 37 °C. Cells were washed twice with PBS, fixed in 3.7% paraformaldehyde (Sigma-Aldrich) for 10 min at room temperature. Afterward, cells were permeabilised with 0.1% Triton-X100 (Sigma-Aldrich) for 15 min and cell cytoskeleton filamentous actin (F-actin) was stained with Alexa Fluor 488-conjugated phalloidin (1:100, Molecular Probes) for 30 min followed by nuclei staining with 4′,6-diamidino-2-phenylindole (DAPI, 1 μg/mL, BD Bioscience) for 10 min. Images of the stained cells were acquired using the Selena S digital imaging system (Logos Biosystems).

#### Apoptosis assay by Flow Cytometry

The apoptotic cell index, i.e. the relative percentage of cells undergoing early and late apoptosis, was assessed by staining the cells with Annexin V conjugated to fluorescein isothiocyanate (FITC) and PI. Early in the apoptotic process, caspases and other proteases cleave the cytoskeleton and the plasma membrane loses its rigidity, due to the deleterious effects in the underlying net of actin. This process disrupts the phospholipid asymmetry, leading to an increase of exposure of phosphatidylserine (PS) on the outer leaflet of plasma membrane^32^. Annexin V binds to PS; still, at this stage, damaged plasma membrane is ion selective for a long time excluding PI (Annexin V-FITC^+^/PI^−^; early apoptotic cells). In later stages of apoptosis, cells enter a blebbing process, leading to a shrunken morphology, and loose the membrane integrity and the ability to exclude PI^32,33^ (Annexin V-FITC^+^/PI^+^; late apoptotic and/or necrotic cells). Normal, viable cells are unstained (V-FITC^−^/PI^−^).

Cells were cultured for 24 h, and then they were exposed to HA-NP suspension or leachate for 1 h and 4 h. After exposure, the levels of apoptosis were determined by staining the cells with Annexin V-FITC Apoptosis Detection Kit (640914, Biolegend), according to the manufacturer’s protocol. Stained cells were immediately evaluated by flow cytometry (FACScalibur cytometer) and the data were analysed using FlowJo software.

#### ROS production

To evaluate the potential of HA-NP of inducing oxidative stress, ROS production was assessed using the DCFDA Cellular Reactive Oxygen Species Detection Assay Kit (Abcam) according with the protocol supplied by the manufacturer. This kit contains a cell permeant reagent 2′,7′- dichlorofluorescin diacetate (DCFDA), which is a fluorogenic dye that measures hydroxyl, peroxyl and other ROS activity within the cell. After the diffusion of DCFDA into the cell, this dye is hydrolysed by cellular esterases to a non-fluorescent compound, which is later oxidized by ROS to a highly fluorescent compound 2′,7′- dichlorofluorescin (DCF) that can be detected using fluorescence spectroscopy^2^. Briefly, 24 h after cell seeding, the medium was discarded and cells were washed with a specific buffer from the kit. Afterwards, buffer was removed and cells were incubated with DCFDA solution for 45 minutes in the dark. After incubation, the DCFDA solution was removed, cells were washed with PBS and the HA-NPs suspension or leachate were added. Tert-butyl hydrogen peroxide (TBHP) in a concentration of 500 µM was used as positive control and non-treated cells were used as negative control. Fluorescence was measured in a microplate reader (SynergyMix, BioTek) after 2, 3 and 4 hours of exposure at 485 nm excitation and 528 nm emission. Results are expressed in terms of relative fluorescence units (RFU).

### The irritation potential by the HET-CAM Assay

HA-NP suspension or leachate were tested for the irritation potential using the chorioallantoic membrane (CAM) assay according to Interagency Coordinating Committee on the Validation of Alternative Methods (ICCVAM) guidelines^34^.

Freshly laid fertilized chicken eggs were incubated horizontally at 37 °C in a 60% humidified atmosphere, using an Octagon Advance incubator (Brinsea) with one hour scheduled rotation. At day 8-post fertilization, a small window was made in the eggshell, under aseptic conditions, to access the CAM beneath. 10 µL of HA-NP samples were placed on the CAM of the chicken embryo in an area delimited by silicon rings, which were randomly distributed and individually positioned on the CAM. For a contact period of 1, 3 and 5 min, the CAM was assessed by visual inspection and photographed with a stereomicroscope attached to a digital camera (Lan Optics), for signs of irritation. The irritation potential was evaluated by the occurrence of specific effects to the membranes and/or vessels (i.e. hyperaemia, haemorrhage, clotting and changes in small vessel diameter) which were interpreted in comparison to a negative (0.9% NaCl) and a positive (0.5 M NaOH) control according to ICCVAM^34^. Time dependent scores for irritancy were assigned semi-quantitatively using a grading system according to the Luepke method, from 0 (no-reaction) to 3 (strong reaction). Each test was carried out in triplicate. Six eggs were used for each test item.

### Statistical analysis

In the biological assays, three independent experiments were performed, each one in triplicate. Results are presented as mean ± standard deviation. The statistical analysis was performed using GraphPad Prism Software version 5.01. The data analysis was done using the one-way analysis of variance (One-way ANOVA) followed by post hoc Tukey test with a significance level of p < 0.05.

## Supplementary information

Supplementary figures 1, 2 and 3
