# Supplementary material for: Nano-hydroxyapatite in oral care cosmetics: characterization and cytotoxicity assessment
Source: Sci Rep. 2019 Jul 30;9:11050. doi: 10.1038/s41598-019-47491-z (PMC6667430; doi:10.1038/s41598-019-47491-z)
Supplement: Supplementary file 1 — Supplementary figures 1, 2 and 3 [file 41598_2019_47491_MOESM1_ESM.pdf]

Nano-hydroxyapatite in oral care cosmetics: characterization and cytotoxicity assessment

Catarina C. Coelho[a,b,c,d,*], Liliana Grenho[e,f], Pedro S. Gomes[e,f], Paulo A. Quadros[d], Maria H. Fernandes[e,f]

[a]*Instituto de Investigação e Inovação em Saúde, U.Porto, Porto, Portugal*

Address: Rua Alfredo Allen, 208, 4200-135 Porto, Portugal

[b]*Instituto de Engenharia Biomédica, U.Porto, Porto, Portugal*

Address: Rua Alfredo Allen, 208, 4200-135 Porto, Portugal

[c]*Faculdade de Engenharia, U.Porto, Porto, Portugal*

Address: Rua Dr. Roberto Frias, s/n, 4200-465 Porto, Portugal

[d]*FLUIDINOVA, S.A., Maia, Portugal*

Address: Rua Engenheiro Frederico Ulrich, 2650, 4470-605 Moreira da Maia, Portugal

[e]*Laboratory for Bone Metabolism and Regeneration, Faculdade de Medicina Dentária, U.Porto, Porto, Portugal*

Address: Rua Dr. Manuel Pereira da Silva, s/n, 4200-393 Porto, Portugal

[f]*LAQV/REQUIMTE, U.Porto, Porto, 4160-007, Portugal*

**Corresponding author (catarina.coelho@i3s.up.pt)*

Supplementary Information

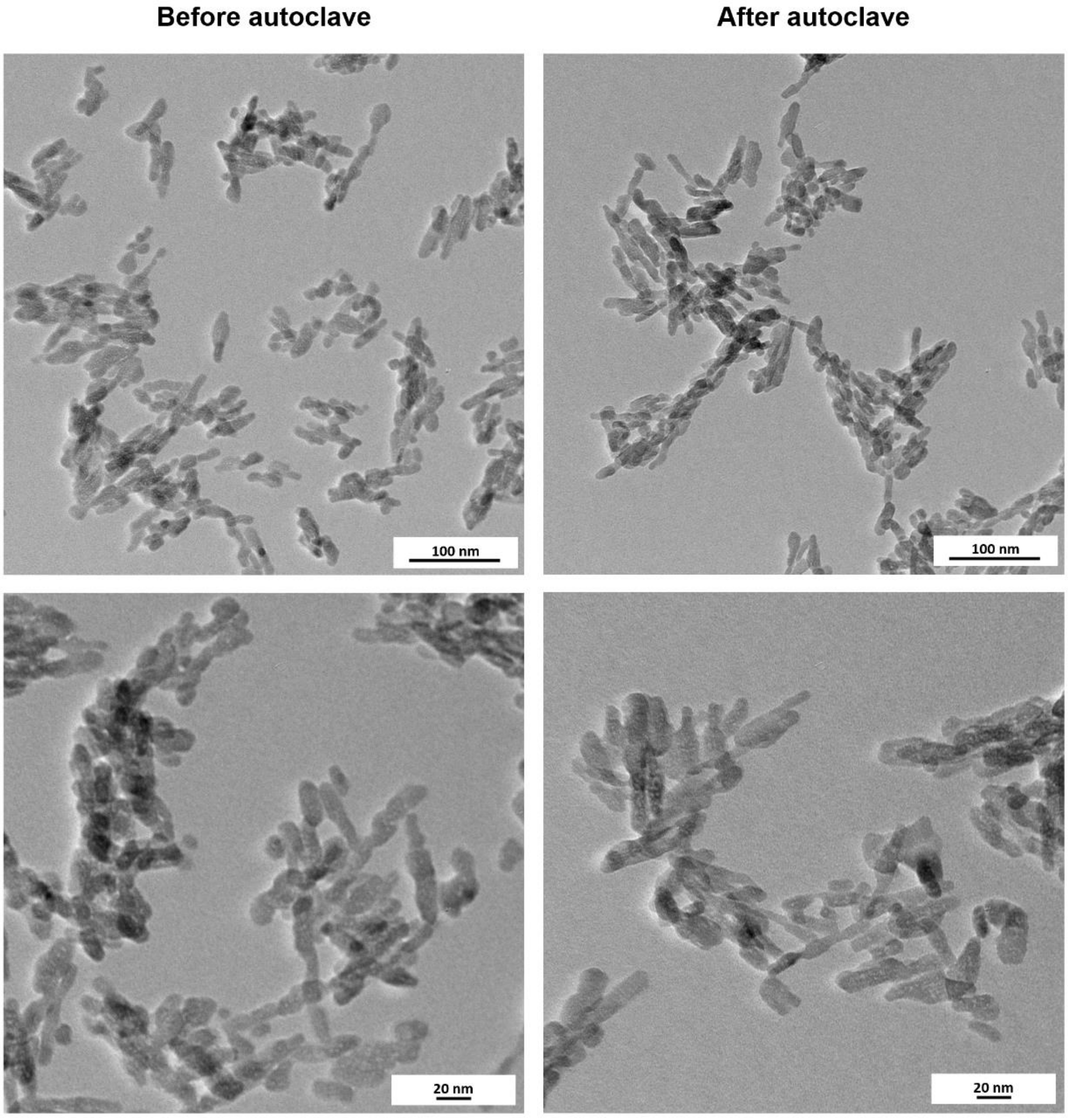

Supplementary Figure 1: TEM images of the hydroxyapatite nanoparticles before and after sterilization using autoclave.

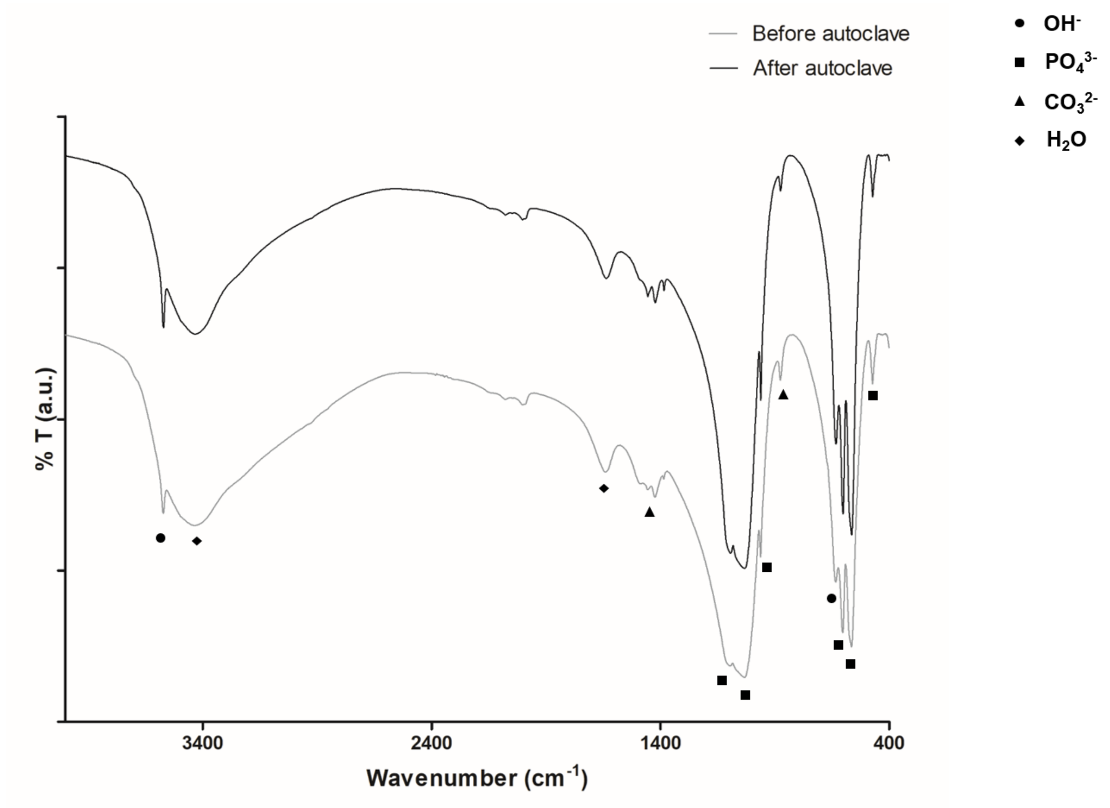

Supplementary Figure 2: FTIR spectra for hydroxyapatite nanoparticles before and after sterilization by autoclave.

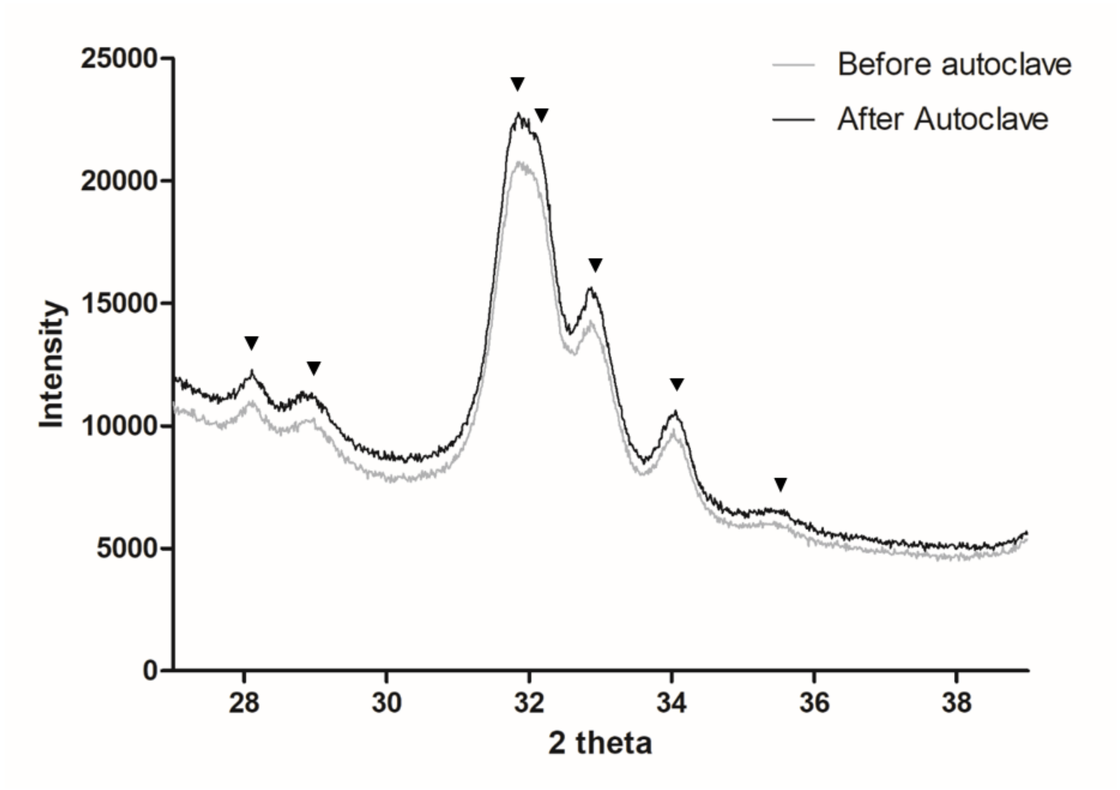

Supplementary Figure 3: XRD patterns obtained for hydroxyapatite nanoparticles before and after sterilization with autoclave.
